# The Chichester Infirmary

**Published:** 1906-12-15

**Authors:** Robert W. Garnham

**Affiliations:** Lieut.-Colonel. Densworth, Chichester


THE CHICHESTER INFIRMARY.

Sir,—I have received a copy of The Hospital [containing an article commenting, as I presume, upon a letter of mine published in our local papers], and for which, if you are the sender, I beg to thank you very much for your courtesy.

I am past eighty-one years of age, and, with my rheumatic hands, do not write as easily as of yore, but I should like to put matters a little more clearly before you, and will do so as quickly as I am able to manage the writing. I should be very glad if the committee of Chichester Infirmary would do as you suggest, and express " a clear statement," but I have not been able at our meetings to obtain any explanation of changes in the statutes, and, as regards the legality of the meetings for the acceptance and subsequent confirmation of alterations, they simply treated my protests as questions of order, and the Chairman decided against me and went on with the business, although, as I stated in my letter, it was admitted that two protests from the hon. solicitor and confirming counsel's opinion had been laid before them.

Their not attending to counsel's opinion was entirely based on not having received with it (from their own solicitor) his case which he last sent up for opinion, but they gave no explanation as to why they ignored the opinion of the solicitors themselves.

Faithfully yours,

Robert W. Garnham, Lieut.-Colonel.

Densworth, Chichester.

H.R.H. The Duke of Connaught, K.G., has become one of the Vice-Patrons of the North London or University College Hospital.

Messrs. Buchanan and Co., Ltd., have had the honour of appointment by Royal warrant to supply Scotch whisky to His Majesty the King of Spain.

